# Why does Russia have such high cardiovascular mortality rates? Comparisons of blood-based biomarkers with Norway implicate non-ischaemic cardiac damage

**DOI:** 10.1136/jech-2020-213885

**Published:** 2020-09-01

**Authors:** Olena lakunchykova, Maria Averina, Tom Wilsgaard, Hugh Watkins, Sofia Malyutina, Yulia Ragino, Ruth H Keogh, Alexander V Kudryavtsev, Vadim Govorun, Sarah Cook, Henrik Schirmer, Anne Elise Eggen, Laila Arnesdatter Hopstock, David A Leon

**Affiliations:** 1 Department of Community Medicine, UiT the Arctic University of Norway, Tromsø, Norway; 2 Department of Laboratory Medicine, University Hospital of North Norway, Tromsø, Norway; 3 Division of Cardiovascular Medicine, Radcliffe Department of Medicine, University of Oxford, Oxford, UK; 4 Wellcome Trust Centre for Human Genetics, University of Oxford, Oxford, UK; 5 Novosibirsk State Medical University, Russian Ministry of Health, Novosibirsk, Russian Federation; 6 Research Institute of Internal and Preventive Medicine, Branch of Institute of Cytology and Genetics, Siberian Branch of the Russian Academy of Sciences, Novosibirsk, Russian Federation; 7 Department of Medical Statistics, London School of Hygiene & Tropical Medicine, London, UK; 8 Department of Innovative Programs, Northern State Medical University, Arkhangelsk, Russian Federation; 9 Federal Research and Clinical Center of Physical-Chemical Medicine of the Federal Medical Biological Agency of Russia, Moskva, Russian Federation; 10 Department of Cardiology, Akershus University Hospital, Lorenskog, Norway; 11 Institute of Clinical Medicine, University of Oslo, Oslo, Norway; 12 Department of Non-communicable Disease Epidemiology, London School of Hygiene & Tropical Medicine, London, UK

**Keywords:** Cardiovascular disease, Epidemiology of cardiovascular disease, Biostatistics, Cohort studies, Epidemiology, Statistics, Epidemiological methods, Pharmacoepidemiology, Health inequalities, Prescribing, Gender, Seasonal, CHD/Coronary heart

## Abstract

**Background:**

Russia has one of the highest rates of mortality from cardiovascular disease (CVD). At age 35–69 years, they are eight times higher than in neighbouring Norway. Comparing profiles of blood-based CVD biomarkers between these two populations can help identify reasons for this substantial difference in risk.

**Methods:**

We compared age-standardised mean levels of CVD biomarkers for men and women aged 40–69 years measured in two cross-sectional population-based studies: Know Your Heart (KYH) (Russia, 2015–2018; n=4046) and the seventh wave of the Tromsø Study (Tromsø 7) (Norway, 2015–2018; n=17 646). A laboratory calibration study was performed to account for inter-laboratory differences.

**Results:**

Levels of total, low-density lipoprotein-, high-density lipoprotein-cholesterol and triglycerides were comparable in KYH and Tromsø 7 studies. N-terminal pro-b-type natriuretic peptide (NT-proBNP), high﻿-sensitivity cardiac ﻿troponin T (hs-cTnT) and high﻿-sensitivity C-reactive protein (hsCRP) were higher in KYH compared with Tromsø 7 (NT-proBNP was higher by 54.1% (95% CI 41.5% to 67.8%) in men and by 30.8% (95% CI 22.9% to 39.2%) in women; hs-cTnT—by 42.4% (95% CI 36.1% to 49.0%) in men and by 68.1% (95% CI 62.4% to 73.9%) in women; hsCRP—by 33.3% (95% CI 26.1% to 40.8%) in men and by 35.6% (95% CI 29.0% to 42.6%) in women). Exclusion of participants with pre-existing coronary heart disease (279 men and 282 women) had no substantive effect.

**Conclusions:**

Differences in cholesterol fractions cannot explain the difference in CVD mortality rate between Russia and Norway. A non-ischemic pathway to the cardiac damage reflected by raised NT-proBNP and hs-cTnT is likely to contribute to high CVD mortality in Russia.

## INTRODUCTION

Russia has one of the highest rates of mortality from cardiovascular disease (CVD) in the world,^1^ although it has been falling since 2005.^2^ The causes of this high CVD mortality are not fully understood. Comparison of blood-based biomarkers and other risk factors in Russia relative to other countries with lower CVD risk should throw light on the likely drivers of these differences in mortality. A small number of such studies have been conducted with blood-based biomarkers restricted to lipid profiles (total cholesterol, low-density lipoprotein (LDL)﻿-cholesterol, high-density lipoprotein (HDL)﻿-cholesterol and triglycerides).^3–^ ^6^ These have generally found no major differences between Russia and other countries.

Biomarkers such as high﻿-sensitivity cardiac ﻿troponin T (hs-cTnT) and N-terminal pro-b-type natriuretic peptide (NT-proBNP) provide information on actual cardiovascular morbidity and are not simply risk predictors. They have been increasingly used in population-based research where they have been shown to be independent predictors of CVD events.^7–^ ^9^ Outside of acute ischaemic cardiac events, hs-cTnT elevation is associated with future risk of heart failure, which is supported by structural and functional studies of the heart.^10^ NT-proBNP is used in diagnostics of heart failure and is predictive of heart failure in population-based cohorts,^11^ along with atrial fibrillation and stroke.^12^ While some controversy exists about the role of high﻿-sensitivity C-reactive protein (hsCRP) in CVDs,^13^ it is associated with coronary heart disease, stroke and vascular death independently of the traditional risk factors.^14^ In fact, studies of large population-based cohorts identified hsCRP, hs-cTnT and NT-proBNP as the blood biomarkers that are the most predictive of cardiovascular events.^15^

In this paper, data from the Know Your Heart (KYH) study (Russia) and the Tromsø ﻿study (Norway) are compared to establish the differences in major cardiovascular biomarkers measured in blood among men and women aged 40–69 years. Norway has a CVD mortality rate approximately eight times lower than that in Russia in this middle-aged group^16^; thus, it provides a good contrast for comparing CVD biomarker levels.

## METHODS

### Study populations

*Know Your Heart (Russia).* A random population-based sample of participants aged 35–69 years (n=5107) stratified by age, sex and district were recruited in the cities of Arkhangelsk and Novosibirsk (Russia).^16^ Trained interviewers recruited and interviewed participants at home to ascertain information about their health, socio-demographic characteristics and lifestyle (51% of approached agreed to participate). Participants were then invited to take part in a health check at an outpatient clinic and 4543 (89%) attended. Our analysis is based on 4046 participants aged 40–69 years who attended the health check and provided a blood sample. The health check included blood pressure measurements, recording of weight and height, a 12-lead ECG and biological sample collection. The additional questionnaire collected data on health problems, lifestyle and medication use. Within 2 hours after venipuncture (non-fasting samples), blood was centrifuged, serum was frozen (−80℃), and analysed in a single batch at the end of the fieldwork in Moscow.^16^

The *Tromsø Study (Norway).* In Tromsø 7, all inhabitants of the municipality of Tromsø aged 40 years and above were invited and 21 083 participated (65%). The subset of 17 646 participants aged 40–69 years was included in our analysis. All participants completed questionnaires and examinations including biological sampling. The questionnaire covered lifestyle, medication use and medical history. A random subsample (5965 participants) attended a second visit. Blood samples (non-fasting) at both visits were processed immediately after collection and the laboratory assays of the biomarkers were performed the same day at the Department of Laboratory Medicine, University Hospital of Northern Norway (ISO certification NS-EN ISO 15 189:2012).

### Study measurements

All participants in KYH and Tromsø 7 with blood sample collected had measured lipid profile (total cholesterol, LDL-cholesterol, HDL-cholesterol, triglycerides), a marker of systemic inflammation (hsCRP) and glycosylated haemoglobin (HbA1c)—Supplementary Table S1. A marker of cardiac damage (hs-cTnT) and a marker of cardiac wall stretch (NT-proBNP) were measured in all KYH participants and in 1403 Tromsø 7 participants who were either selected randomly (81%) to attend the second visit or were invited because of their previous participation in the sixth wave of the Tromsø study. The characteristics of those in Tromsø study with measured cardiac biomarkers are very similar to that of the total study sample (Supplementary Table S2).

10.1136/jech-2020-213885.supp1 Supplementary data

Body mass index (BMI) was calculated as weight (kilograms) divided by height (metres) squared. Mean systolic and diastolic blood pressure was calculated as the mean of second and third measurements. Waist circumference (WC) was measured at the narrowest part of the trunk in KYH, while in Tromsø 7, WC was measured at the umbilicus level. To ensure WC was comparable between the two studies, WC in Tromsø 7 was converted to the narrowest waist using a conversion equation.^17^ Waist-to-hip ratio (WHR) was calculated by dividing WC by hip circumference. Smoking status was categorised as current smokers, ex-smokers and never-smokers. For current smokers, the number of cigarettes smoked was specified as 1–10/day, 11–20/day and >20/day. Education level was classified into three categories: primary/secondary, upper secondary and tertiary. Diabetes was defined as HbA1c concentration above 6.5%, or self-report of diabetes, or use of medication with ATC-code A10 (antidiabetics) according to the Anatomical Therapeutic Chemical (ATC) classification.^18^ Lipid-lowering drugs use was determined according to recorded medications coded to the ATC classification as C10 (lipid﻿-modifying agents) or self-reported use.

The pre-existing coronary heart disease was determined as evidence of previous myocardial infarction (MI) on ECG, self-report of MI or grade 2 angina pectoris. ECGs from both studies were coded according to the Minnesota code (MC 1.1–1.3)^19^ using the same semi-automated system. Grade 2 angina was determined using the Rose ﻿Angina Questionnaire (short version).^20^

### Calibration of laboratory data

Differences in the laboratory procedures in KYH and Tromsø 7 bring the potential for systematic differences in biomarker measurements between the two sites due to measurement error. This was addressed by a recalibration study with split sample testing (Supplementary Methods M1, Supplementary Tables S3–S5, Supplementary Figures S1–S10). For that purpose, 100 serum samples and 50 whole blood samples from KYH participants were re-assayed in both the laboratories in Moscow and Tromsø. The paired measurements were analysed using Deming regression to derive the calibration equations.

### Statistical analysis

Mean biomarker levels among men and women were compared having age-standardised to the 2013 Standard European Population. Biomarkers with skewed distributions (triglycerides, hsCRP, hs-cTnT, NT-proBNP) were ln-transformed before analysis and geometrical means were presented. Multivariable linear regression was used to assess if the differences in mean biomarker levels in the two studies could be explained by differences in age (Model1), smoking prevalence, BMI, WHR, blood pressure, diabetes, education level (in addition to age) (Model 2) and use of lipid-lowering drugs (in addition to variables in Model 2) (Model 3). For triglycerides, models were also adjusted for the fasting status. The regression models for hs-cTnT and NT-proBNP were repeated for study participants without previous MI or grade 2 angina. For the regression modelling, data from participants with complete information on all the covariates were used. For skewed biomarkers, the regression coefficients were back-transformed to be interpreted as a per cent difference between studies. Based on finding evidence of an interaction between age and study, the differences in biomarkers between studies were presented separately for 40–54 and 55–69 year olds.

All analyses were done using recalibrated biomarkers (Supplementary Table S5). To account for uncertainty in the estimation of the calibration coefficients in the subsequent comparative analysis, we used a ‘double-bootstrap’ approach, verified using a simulation study (Supplementary Methods M2, Supplementary Tables S6–S7), to obtain 95% CIs for the regression coefficients. Statistical analysis was performed using R version 3.6.0 and SAS software 9.4 (SAS Institute Inc., Cary, NC, USA).

## RESULTS

Descriptive characteristics presented in table 1 show that men in KYH were on average older, had higher blood pressure and lower BMI, and a higher proportion were current smokers, and had diabetes, compared with men in Tromsø 7. Women in KYH were on average older, had higher blood pressure and BMI, a higher proportion had diabetes, and a lower proportion were current or previous smokers, compared with women in Tromsø 7 (table 1). The similar proportion of participants reported using lipid-lowering drugs that could be identified by ATC code in KYH and Tromsø 7; however, self-reported use of lipid-lowering drugs was higher in KYH (table 1).

**Table 1:** Characteristics of the study sample (participants aged 40–69 years with blood sample collected): KYH (N=4046) and Tromsø 7 (N=17 555)

	Men		Women	
	KYH†	Tromsø 7‡	KYH	Tromsø 7
Age, mean (SD)	56.2 (8.5)	53.8 (8.5)	55.9 (8.7)	53.6 (8.4)
SBP, mean (SD)	138.6 (19.8)	130.8 (17.1)	129.9.0 (19.6)	123.4 (18.7)
DBP, mean (SD)	86.5 (11.3)	78.8 (9.7)	81.3 (11)	72.6 (9.6)
Hypertension§	1102 (63.6)	3462 (41.5)	1337 (56.0)	2759 (29.7)
Smoking, N (%)				
Current smoker >20/day	110 (6.5)	47 (0.6)	21 (0.9)	21 (0.2)
Current smoker 11–20/day	376 (22.2)	477 (5.8)	154 (6.6)	353 (3.9)
Current smoker 1–10/day	136 (8.0)	1083 (13.3)	200 (8.6)	1399 (15.3)
Ex-smoker	640 (37.8)	3329 (40.7)	370 (15.8)	4109 (44.8)
Never smoked	432 (25.5)	3226 (39.5)	1595 (68.1)	3279 (35.8)
BMI, mean (SD)	27.7 (4.8)	27.9 (4.0)	28.9 (6.2)	26.8 (4.9)
WHR, mean (SD)	0.95 (0.07)	0.94 (0.07)	0.85 (0.08)	0.79 (0.07)
HbA1c ≥6.5%, N (%)	195 (11.6)	404 (4.9)	262 (11.3)	264 (2.9)
Use of diabetes medication, N (%)	76 (4.5)	350 (4.2)	170 (7.3)	271 (2.9)
Diabetes, N (%)	217 (12.8)	515 (6.2)	353 (15.1)	393 (4.3)
Lipid-lowering drugs (ATC code C10 and/or self-report), N (%)	266 (15.7)	1090 (13.1)	468 (20.0)	837 (9.1)
Lipid-lowering drugs (ATC code C10), N (%)	169 (10.8)	913 (10.9)	212 (9.9)	720 (7.74)
Education level				
Primary/secondary	147 (8.7)	1624 (19.6)	134 (5.7)	1699 (18.36)
Upper secondary	878 (51.7)	2570 (31.0)	1280 (54.6)	2409 (26.0)
Tertiary	675 (39.7)	4108 (49.5)	932 (39.7)	5145 (55.6)
Pre-existing coronary heart disease	238 (14.0)	471 (5.7)	259 (11.0)	215 (2.3)

†Missing data in KYH: SBP/DBP—334 (8.3%), smoking—12 (0.3%), BMI—12 (0.3%), WHR—2 (0.1%), diabetes—18 (0.4%), HbA1c—51 (1.3%), diabetes medication—419 (10.4%).

‡Missing data in Tromsø 7: SBP/DBP—45 (0.3%), smoking—273 (1.6%), BMI—41 (0.2%), WHR—65 (0.4%), HbA1c—135 (0.8%).

§Hypertension was defined as SBP >140 mmHg and/or DPB >90 mmHg and/or use of antihypertensive medication (ATC codes C02 (antihypertensives), C03 (diuretics), C07 (beta-blocking agents), C08 (calcium channel blockers), or C09 (agents operating on the renin-angiotensin system) and/or self-reported use.

ATC, Anatomical Therapeutic Chemical; BMI, body mass index; DBP, diastolic blood pressure; KYH, Know Your Heart; SBP, systolic blood pressure; WHR, waist-to-hip ratio.

The age-standardised means of CVD biomarkers are compared in table 2. The geometric means for hs-cTnT, NT-proBNP and hsCRP were significantly higher in KYH compared with Tromsø 7 among both men and women. It is notable that KYH had a higher proportion of participants with detectable hs-cTnT: 98.6% compared with 64.4% in Tromsø 7.

**Table 2:** Age-standardised means† of CVD biomarkers in KYH and Tromsø 7

	KYH		Tromsø 7		P value for difference
	N	Mean (95% CI)	N	Mean (95% CI)	
Men					
Total cholesterol (mmol/L)	1700	5.26 (5.21, 5.31)	8302	5.46 (5.44, 5.48)	<0.001
HDL-cholesterol (mmol/L)	1700	1.34 (1.32, 1.36)	8301	1.37 (1.36, 1.38)	0.002
LDL-cholesterol (mmol/L)	1700	3.44 (3.39, 3.48)	8302	3.70 (3.67, 3.72)	<0.001
Triglycerides (mmol/L)‡	1700	1.45 (1.41, 1.49)	8302	1.54 (1.52, 1.55)	<0.001
hsCRP (mg/L)‡	1700	1.42 (1.35, 1.49)	8302	1.06 (1.04, 1.08)	<0.001
NT-proBNP (pg/ml)‡	1700	54.7 (52.4, 57.2)	650	35.3 (32.5, 38.4)	<0.001
hs-cTnT (ng/L)‡	1700	7.59 (7.42, 7.77)	645	5.23 (5.01, 5.46)	<0.001
Women					
Total cholesterol (mmol/L)	2346	5.50 (5.46, 5.54)	9253	5.53 (5.51, 5.55)	0.138
HDL-cholesterol (mmol/L)	2346	1.61 (1.59, 1.63)	9253	1.72 (1.71, 1.73)	<0.001
LDL-cholesterol (mmol/L)	2346	3.54 (3.50, 3.58)	9253	3.56 (3.54, 3.58)	0.569
Triglycerides (mmol/L)‡	2346	1.30 (1.27, 1.32)	9253	1.18 (1.16, 1.19)	<0.001
hsCRP (mg/L)‡	2346	1.37 (1.32, 1.43)	9253	1.03 (1.01, 1.05)	<0.001
NT-proBNP (pg/mL)‡	2342	71.0 (68.9, 73.1)	762	56.5 (53.3, 59.8)	<0.001
hs-cTnT (ng/L)‡	2342	5.93 (5.83, 6.02)	758	3.58 (3.47, 3.69)	<0.001

†Standardised to the Standard European Population 2013.

‡Geometric means are presented.

CVD, cardiovascular disease; HDL, high-density lipoprotein, hsCRP, high-sensitivity C-reactive protein; hs-cTnT, high-sensitivity cardiac troponin T; KYH, Know Your Heart; LDL, low-density lipoprotein; NT-proBNP, N-terminal pro-b-type natriuretic peptide.

Table 3 shows the conditional differences in mean biomarker levels between the studies from three regression models. The age-adjusted model shows that men and women in KYH had slightly lower LDL- and HDL-cholesterol than in Tromsø 7, while triglyceride levels in women were higher in KYH. Adjustment for smoking, BMI, WHR, blood pressure, diabetes, education, and use of lipid-lowering drugs use had little effect on these differences (Model 2 and Model 3).

**Table 3:** Differences† in mean biomarker levels in KYH vs Tromsø 7 adjusted for CVD risk factors

	N	Model 1 (adjusted for age)	Model 2 (adjusted for age, smoking, BMI, WHR, SBP, DBP, diabetes, education)	Model 3 (adjusted for age, smoking, BMI, WHR, SBP, DBP, diabetes, education, lipid-lowering drugs)
Men				
Total cholesterol	9669	−0.22 (−0.29, −0.1)	−0.31 (−0.39, −0.19)	−0.30 (−0.38, −0.17)
HDL	9669	−0.05 (−0.07, −0.02)	−0.05 (−0.07, −0.02)	−0.05 (−0.07, −0.02)
LDL	9679	−0.26 (−0.34, −0.22)	−0.32 (−0.41, −0.28)	−0.31 (−0.39, −0.27)
Triglycerides‡§	9454	0.03 (0.00, 0.07)	0.02 (−0.01, 0.06)	0.02 (−0.01, 0.06)
hsCRP‡	9669	0.29 (0.23, 0.35)	0.16 (0.10, 0.22)	0.17 (0.11, 0.22)
NT-proBNP‡	2192	0.44 (0.36, 0.53)	0.37 (0.27, 0.47)	0.37 (0.27, 0.46)
hs-cTnT‡	2197	0.36 (0.31, 0.40)	0.37 (0.32, 0.42)	0.37 (0.32, 0.42)
Women				
Total cholesterol	11 189	−0.07 (−0.15, 0.04)	−0.13 (−0.21, −0.01)	−0.09 (−0.17, 0.03)
HDL	11 189	−0.13 (−0.16, −0.11)	−0.02 (−0.04, 0.01)	−0.02 (−0.04, 0.01)
LDL	11 189	−0.03 (−0.10, 0.01)	−0.13 (−0.21, −0.09)	−0.09 (−0.17, −0.05)
Triglycerides‡	10 859	0.10 (0.07, 0.12)	0.03 (0.00, 0.06)	0.03 (0.00, 0.06)
hsCRP‡	11 189	0.31 (0.26, 0.35)	0.04 (−0.01, 0.10)	0.05 (0, 0.11)
NT-proBNP‡	2876	0.27 (0.21, 0.33)	0.33 (0.25, 0.39)	0.32 (0.24, 0.38)
hs-cTnT‡	2880	0.52 (0.48, 0.55)	0.49 (0.45, 0.53)	0.49 (0.45, 0.53)

†Values in KYH minus those in Tromsø 7 and 95% CIs, all models based on cases without missing data on adjustment variables.

‡Analysis is based on ln-transformed values.

§The models for triglycerides were additionally adjusted for fasting time because of differences in mean fasting time in the two studies. Fasting time was recorded from participants' self-report.

BMI, body mass index; CVD, cardiovascular disease; DBP, diastolic blood pressure; HDL, high-density lipoprotein, hsCRP, high-sensitivity C-reactive protein; hs-cTnT, high-sensitivity cardiac troponin T; KYH, Know Your Heart; LDL, low-density lipoprotein; NT-proBNP, N-terminal pro-b-type natriuretic peptide; systolic blood pressure; WHR, waist-to-hip ratio.

In the age-adjusted model, hsCRP in KYH was 33.3% (95% CI 26.1% to 40.8%) higher in men and 35.6% (95% CI 29.0% to 42.6%) higher in women compared with Tromsø 7 (untransformed coefficients in table 3). The corresponding values for NT-proBNP were 54.1% (95% CI 41.5% to 67.8%) and 30.8% (95% CI 22.9% to 39.2%), and for hs-cTnT—42.4% (95% CI 36.1% to 49.0%) and 68.1% (95% CI 62.4% to 73.9%). There was substantial attenuation of the differences in hsCRP due to adjustment by smoking, BMI, WHR, blood pressure, diabetes and education, but there remained evidence for differences between the two studies (Model 2). For hs-cTnT and NT-proBNP, adjustment did not change the estimate of the mean difference.

The differences in hs-cTnT and NT-proBNP remained when the analysis was restricted to participants without previous MI, or grade 2 angina (table 4).

**Table 4:** The difference in mean levels of NT-proBNP and hs-cTnT in KYH compared with Tromsø 7, adjusted for age.

	Without coronary heart disease		With coronary heart disease	
	N	Mean difference (95% CI)	N	Mean difference (95% CI)
Men				
NT-proBNP†	1913	0.42 (0.33, 0.51)	279	0.34 (−0.02, 0.68)
hs-cTnT†	1918	0.35 (0.30, 0.40)	279	0.35 (0.17, 0.52)
Women				
NT-proBNP†	2594	0.24 (0.18, 0.30)	282	0.49 (0.13, 0.81)
hs-cTnT†	2598	0.52 (0.49, 0.55)	282	0.38 (0.12, 0.58)

Analysis is stratified by pre-existing coronary heart disease (ECG or self-reported MI, grade 2 angina).

†Analysis is based on ln-transformed values.

hs-cTnT, high-sensitivity cardiac troponin T; KYH, Know Your Heart; MI, myocardial infarction; NT-proBNP, N-terminal pro-b-type natriuretic peptide.

The differences in biomarker levels between the two studies differed by age group (table 5). For most biomarkers, study differences were larger in women aged 55–69 years than 40–54 years. Among men, differences in hsCRP were more pronounced in the older age group (55–69 years), while differences in total and LDL-cholesterol were larger in younger men (40–54 years).

**Table 5:** Age-stratified differences† in mean biomarker levels in KYH compared with Tromsø 7 by sex, adjusted for age (within strata)

	Men			Women		
	40–54 years old	55–69 years old	P value for interaction	40–54 years old	55–69 years old	p-value for interaction
Total cholesterol	−0.27 (−0.36, −0.19)	−0.17 (−0.25, −0.09)	0.077	0.05 (−0.01, 0.12)	−0.18 (−0.25, −0.12)	<0.001
HDL cholesterol	−0.01 (−0.04, 0.02)	−0.08 (−0.11, −0.05)	0.002	−0.03 (−0.06, 0.00)	−0.22 (−0.25, −0.19)	<0.001
LDL cholesterol	−0.33 (−0.41, −0.25)	−0.20 (−0.27, −0.13)	0.02	0.01 (−0.06, 0.07)	−0.06 (−0.12, 0.00)	0.157
Triglycerides‡§	−0.01 (−0.06, 0.04)	0.07 (0.02, 0.11)	0.012	0.12 (0.09, 0.16)	0.19 (0.15, 0.22)	0.016
hsCRP‡	0.18 (0.10, 0.25)	0.38 (0.31, 0.45)	<0.001	0.19 (0.13, 0.26)	0.41 (0.34, 0.47)	<0.001
NT-proBNP	0.33 (0.19, 0.48)	0.49 (0.39, 0.59)	0.081	0.07 (−0.03, 0.17)	0.40 (0.32, 0.47)	<0.001
hs-cTnT‡	0.35 (0.27, 0.43)	0.36 (0.31, 0.42)	0.788	0.44 (0.38, 0.49)	0.57 (0.53, 0.61)	<0.001

†Values in KYH minus those in Tromsø 7.

‡Analysis is based on ln-transformed values.

§The models for triglycerides were additionally adjusted for fasting time because of differences in mean fasting time in two studies.

HDL, high-density lipoprotein; hsCRP, high-sensitivity C-reactive protein; hs-cTnT, high-sensitivity cardiac troponin T; KYH, Know Your Heart; LDL, low-density lipoprotein; NT-proBNP, N-terminal pro-b-type natriuretic peptide.

### Sensitivity analysis

As hs-cTnT assays are known to show appreciable imprecision at the low values seen in the general population,^21^ we conducted a sensitivity analysis using logistic regression with hs-cTnT categorised into values below and above the top quintile in this study distribution (men—11 ng/L, women—8.07 ng/L). The results were consistent with hs-cTnT analysed as a continuous outcome (Supplementary Tables S8–S9). Adjustment for lipid-lowering drugs based only on ATC codes in the regression model produced similar results to the main analysis which defined ﻿lipid-lowering drugs based on ATC code and self-reported use.

## DISCUSSION

This comparison study shows that, after adjustment for sex and age, the lipid profile was comparable in KYH (Russia) and in Tromsø 7 (Norway) despite the much higher cardiovascular mortality in Russia. In contrast, biomarkers of cardiac damage have higher concentrations in KYH than in Tromsø 7 even after excluding participants with previous coronary artery disease. These results are not explained by the higher prevalence of hypertension and smoking in Russia, suggesting that mechanisms in addition to coronary heart disease contribute to cardiovascular mortality in Russia.

### Cholesterol fractions

All cholesterol fractions were slightly lower in KYH than in Tromsø 7 among both men and women, although the magnitude of this difference was small and would not translate into large differences in risk of vascular events. This is notable given that Russia has one of the highest CVD mortality rate and a large proportion of CVD death in country’s mortality statistics are attributed to coronary heart disease. These findings are consistent with previous studies comparing lipid levels in Russia with other countries, concluding that blood lipid profiles were similar in Russia and Western countries.^3–^ ^6,^ ^22–^ ^24^

The differences in cholesterol measures between studies are not explained by differences in prevalence of classic risk factors and use of lipid-lowering drugs.

### NT-proBNP

Levels of the cardiac wall stretch biomarker NT-proBNP were higher in KYH compared with Tromsø 7 among both men and women. The differences were not explained by classic CVD risk factors (blood pressure, smoking, BMI, WHR, diabetes). Among women, we found difference between studies only in the older age group (55–69 years old).

Elevated NT-proBNP is a biomarker of cardiac dysfunction related to several pathological processes in the cardiovascular system: heart failure,^25^ atrial fibrillation^26^ and stroke.^12^ We suggest that elevated NT-proBNP in KYH compared with Tromsø 7 may be explained by higher heart damage due to non-ischaemic pathways to heart disease. Although heart damage and the development of chronic heart failure can be facilitated by MI or stable coronary heart disease, our conclusions were robust after exclusion of participants with a history of coronary heart disease.

### High-sensitivity cardiac troponin T

Similar to NT-proBNP, we found higher mean levels of hs-cTnT in KYH compared with Tromsø 7 among both men and women. This was not explained by a different prevalence of classic CVD risk factors (smoking, BMI, WHR, blood pressure, diabetes), but among women, the difference was more pronounced in the older age group (55–69 years old). Our study is the first to measure hs-cTnT in a general population in Russia. Several studies in the US and Western Europe used hs-cTnT measurements in population samples free of known CVD to predict future CVD.^15 27^ High hs-cTnT was recognised as an indicator of heart failure rather than ischaemic damage.^9 10^ Biochemical evidence of myocyte injury was associated with subsequent imaging evidence of replacement fibrosis both in the sample of asymptomatic individuals^9^ and in symptomatic non-ischaemic heart disease populations.^28–^ ^30^ Even in patients with chronic coronary artery disease, hs-cTnT was associated with death and heart failure but not MI.^31^ It is notable that exclusion of participants with pre-existing coronary heart disease in our study did not change the estimates of the differences in hs-cTnT substantially, neither did adjustment for hypertension and smoking.

### High-sensitivity C-reactive protein

This marker of systemic inflammation was higher in KYH than in Tromsø 7. The differences are of similar magnitude among men and women, are more pronounced in older age among men and are appreciably attenuated by adjustment for classical CVD risk factors (smoking, BMI, WHR, blood pressure, diabetes). Several previous studies have investigated predictors of increased hsCRP levels in Russian populations but did not report mean levels or systematically compare them with western studies.^6 32^

Raised levels of hsCRP have been found to be predictive of future CVD events^14 33^ and were associated with coronary plaque burden^34^ and atherosclerosis^35^; however, the relationship is not considered to be causal.^13^ Low-grade elevation of hsCRP is non-specific and may reflect exposure to pro-inflammatory influences including smoking, particulate air pollutants, aspects of diet, medications, oral cavity health, obesity and metabolic syndrome.^35^ While elevated hsCRP levels in KYH indicate higher general inflammatory status in the participants, this may reflect both atherosclerosis and higher prevalence of CVD risk factors, like obesity and smoking. Although this study does not permit inferences about the prevalence of atherosclerosis, elevated hsCRP may indicate greater risk of future CVD outcomes in the Russian sample.

### Strengths and limitations

We analysed biomarker levels in recently obtained population-based samples of men and women within the same age range in the two studies. Similar methodology was used for data and sample collection. A key strength is that a calibration study was done to ensure the comparability of the laboratory essays for biomarkers. Furthermore, an innovatory approach to calculate CIs of the regression coefficients obtained using calibrated measures was developed to ensure 95% coverage.

Because the study was conducted in three cities, and response rates in KYH were not optimal, we should be cautious to generalise the findings to the whole of Norway and Russia. The age distribution of the populations of Novosibirsk and Arkhangelsk was similar to the national average in both cities.^16^ Tromsø and Novosibirsk have higher proportion of population with higher education compared with respective national averages.^16 36^ However, it should be noted that the selected locations have CVD mortality rates that are similar to the national averages.^16^

Considering the ongoing changes in cardiovascular mortality in Russia, there are many other factors that may explain recent reduction, including improvements in treatment for acute CVD events.^2^ However, in this paper, we were focusing on circulating biomarkers in the general population rather than particular high-risk groups.

## CONCLUSIONS

By comparing the blood biomarker profiles in comparable population-based studies conducted in Russia and Norway, the latter a country with much lower CVD mortality rates, we attempted to identify the distinguishing features of CVD epidemic in contemporary Russia that make it unique to the rest of the world. We have found the evidence that non-ischaemic pathways beyond lipid-related mechanisms may take a significant share of CVD morbidity in Russia. The higher levels of NT-proBNP and hs-cTnT in Russia may indicate that this population is at higher risk of dilated cardiomyopathy, heart failure, atrial fibrillation and cardioembolic stroke. Very minor differences in lipid levels are not enough to explain the much high mortality due to coronary heart events in Russia compared with Norway. However, higher pro-inflammatory status reflected by hsCRP and contribution of higher levels of hypertension, BMI and WHR (among women); smoking (among men); and diabetes are very likely to contribute to explaining the high coronary heart disease mortality in Russia.

To further explore heart damage, more in-depth characterisation of heart structure and function with echocardiography and carotid ultrasound is required. Exploration of alcohol use as a potential explanation of biomarker differences should be a potential future research direction.^37^

The results of this study are important from a prevention perspective. As we suggest a substantive proportion of CVD in Russia occurring due to non-ischaemic pathways, additional efforts are needed to detect and treat people with early structural and functional changes in the heart.

**What is already known on this subject**

- Russia has one of the highest rates of mortality from cardiovascular disease (CVD) in the world with the reasons for that not fully understood. A small number of studies measured blood-based biomarkers of CVD in Russia but included only lipid profiles. Comparison of lipid profiles to other countries did not find major differences.

**What this study adds**

- Levels of total cholesterol, low-density lipoprotein cholesterol and high-density lipoprotein cholesterol were similar in two population-based studies conducted in Russia and Norway: Know Your Heart and Tromsø 7. This finding is paradoxical given high cardiovascular mortality rates in Russia. However, markers of cardiac damage and general inflammation were considerably higher in Russian compared with Norwegian study. Non-ischaemic pathways to cardiac damage reflected by raised NT-proBNP and hs-cTnT are likely to contribute to high CVD mortality in Russia.
