# Supplementary material for: Why does Russia have such high cardiovascular mortality rates? Comparisons of blood-based biomarkers with Norway implicate non-ischaemic cardiac damage
Source: J Epidemiol Community Health. 2020 Sep 1;74(9):698–704. doi: 10.1136/jech-2020-213885 (PMC7577103; doi:10.1136/jech-2020-213885)
Supplement: Supplementary data [file jech-2020-213885s001.pdf]

Supplementary material

Why does Russia have such high cardiovascular mortality rates? Comparisons of blood-based biomarkers with Norway implicate non-ischemic cardiac damage

Contents

Supplementary Table S1. The list of the analytic methods used for the analysis of biomarkers in Know Your Heart and Tromsø study……………………………………………………….2

Supplementary Table S2. Differences in main study variables between Tromsø 7 study participants with NT-Pro-BNP measured (N=1403) and rest of Tromsø 7 study participants, Visit 1 (N=16243) in age group 40-69 years, adjusted for age and sex……………………….3

Supplementary Methods M1. Recalibration of Blood Biomarker Measurements in Know Your Heart Study for Comparisons with Tromsø 7 study…………………………………………..4

Supplementary Methods M1. Simulation Study……………………………………………..22

Supplementary Table S8. Age-standardized proportion of participants with hs-cTnT value above top quantile of distribution in KYH and Tromsø 7……………………………………26

Supplementary Table S9. The odds of hs-cTnT being in the top quantile of distribution in KYH study compared to Tromsø 7, explained by adjustment for classical CVD risk factors: smoking, BMI, WHR, SBP and DBP, diabetes, education…………………………………..26

Supplementary Table S1. The list of the analytic methods used for the analysis of biomarkers in Know Your Heart and Tromsø study

	Method Lytech (Know Your Heart)	Instrument	Inter-essay CV (concentration - %)	Method UNN (Tromsø 7)	Instrument	Inter-essay CV (concentration - %)	Biological sample
Total cholesterol	Enzymatic Colour Test	AU 680 Chemistry System / Beckman Coulter	3.88 mmol/L - 1.6%	Enzymatic colorimetric test	Cobas 8000 / Roche	4.8 mmol/L - 1.1%	Serum
HDL cholesterol	Enzymatic Colour Test	AU 680 Chemistry System / Beckman Coulter	0.96 mmol/L - 1.61%	Homogeneous enzymatic colorimetric test	Cobas 8000 / Roche	1.3 mmol/L - 1.6%	Serum
LDL cholesterol	Enzymatic Colour Test	AU 680 Chemistry System / Beckman Coulter	4.31 mmol/L - 4.26%	Homogeneous enzymatic colorimetric test	Cobas 8000 / Roche	3.1 mmol/L – 0.77%	Serum
Triglycerides	Enzymatic Colour Test	AU 680 Chemistry System / Beckman Coulter	1.63 mmol/L - 5.6%	Enzymatic colorimetric test	Cobas 8000 / Roche	1.5 mmol/L - 1.37%	Serum
High sensitivity CRP	Immuno-turbidimetric Test	AU 680 Chemistry System / Beckman Coulter	14.52 mg/L - 2.32%	Particle enhanced immunoturbidimetric assay.	Cobas 8000 / Roche	1.03 mg/L – 5.07%	Serum
HBA1c (Glycated haemoglobin)	Immuno-turbidimetric Test	AU 680 Chemistry System / Beckman Coulter	3.88%	Capillary electrophoresis	Capillarys 3 tera	<3%	Whole blood (EDTA)
Hs Troponin T	Electrochemi-luminescence Immunoassay	Cobas e411 / Roche	136 ng/L - 8.23%	Electrochemiluminescence Immunoassay	Cobas 8000 / Roche	12 ng/L - 6.3%	Serum
Nt-Pro-BNP	Electrochemiluminescence Immunoassay	Cobas e411 analyser / Roche	92.85 pg/ml - 8.15%	Electrochemiluminescence Immunoassay	Cobas 8000 / Roche	238 pg/ml - 4.2%	Serum

Supplementary Table S2. Differences in main study variables between Tromsø 7 study participants with NT-Pro-BNP measured (N=1403) and rest of Tromsø 7 study participants, Visit 1 (N=16243) in age group 40-69 years, adjusted for age and sex

	NT-Pro-BNP measured (N=1403)	Visit 1 participants (N=16243)	P-value
Total cholesterol (mmol/L), mean (sd)	5.47 (1.05)	5.50 (1.04)	0.313
HDL-cholesterol (mmol/L), mean (sd)	1.59 (0.50)	1.55 (0.47)	0.008
LDL- cholesterol (mmol/L), mean (sd)	3.58 (0.99)	3.62 (0.97)	0.124
Triglyserids, (mmol/L), GM	1.29	1.34	0.027
CRP, (mmol/L), GM	1.04	1.04	0.822
BMI, mean (sd)	27.1 (4.45)	27.3 (4.58)	0.116
Waist to hip ratio, mean (sd)	0.86 (0.10)	0.86 (0.10)	0.165
SBP, mean (sd)	127 (19.0)	127 (18.2)	0.373
DBP, mean (sd)	74.8 (9.92)	75.6 (10.1)	0.004
Education less than college level, % (N)	49.5 (708)	50.0 (7546)	0.462
Current smoker, % (N)	19.4 (266)	19.6 (3127)	0.740
Diabetes, % (N)	4.9 (76)	5.3 (814)	0.191
Lipid lowering medication, % (N)	10.2 (222)	10.2 (1716)	0.832
MI detected on ECG, % (N)	4.6 (56)	4.6 (175)	0.840
Heart failure (self-report), % (N)	1.7 (27)	1.4 (155)	0.102
Heart attack (self-report), % (N)	2.7 (45)	2.7 (334)	0.929
Grade 2 angina, % (N)	0.8 (16)	0.8 (138)	0.713

Supplementary Methods M1

Recalibration of Blood Biomarker Measurements in Know Your Heart Study for Comparisons with Tromsø 7 study.

Background

Comparisons of biomarker data obtained in different studies may be biased due to the differences in pre-analytic and analytic stages in the laboratory. The similarity of pre-analytic stage has to be ensured during study setup, while the analytic stage bias may be controlled by a calibration study where measurements of one of the studies are recalibrated to the measurements made in another study. In the situation of multicentre or longitudinal studies with laboratory measurements recalibration is needed to correct for laboratory differences in time or space (assay type, assay manufacturer, analytic platform) [1].

The intrinsic quality of a manufacturer's assay or test system might be confounded by the laboratory using the system [2]. An investigation of the comparability of assays produced by different manufacturers showed that assays sometimes do not meet the optimal bias limits and there are considerable calibration differences between manufacturers/assays [2]. Even small biases that occur with use of different assays, instruments or procedures may have considerable implications for the conclusions of research studies and affect comparability in the research setting [3]. At the population level, small, systematic differences shift the entire distribution of a biomarker, resulting in biased estimates of mean values and prevalence of a condition under study defined in terms of a cut-off level [1]. Epidemiologic studies must carefully assess the calibration and reproducibility of their biomarker measurements to ensure equivalence across study sites.

The goal of this calibration study is to derive a calibration equation that reflects the bias (systematic difference) in the measurement of biomarkers in Know Your Heart (KYH) relative

to Tromsø 7 study due to the laboratory analytic stage. The University Hospital of Northern Norway (UNN) Department of Laboratory Medicine was assigned as the «reference laboratory». Representative samples of properly stored vials of serum and blood samples from Know Your Heart study were re-measured there.

Methods

Eight analytes were included into the calibration study: total cholesterol, high density lipoprotein (HDL) cholesterol, low density lipoprotein (LDL) cholesterol, triglycerides, high sensitivity C-reactive protein (hsCRP), HbA1c (Haemoglobin A1c), high sensitivity cardiac Troponin T (hs-cTnT) and N-terminal pro-B-type natriuretic peptide (NT-proBNP). We obtained stratified random sampling of 102 KYH study participants on the basis of 3 age groups (35–46, 47–58, 59–69) and genders (male/female). For calibration of HbA1c measurements, 50 whole blood samples were selected using uniform sampling procedure. All 102 serum and 50 whole blood samples were split and reassayed at both Lytech laboratory (Russia) and UNN (Norway) in December 2018. The type of the laboratory assay, platform and the coefficient of variation for both laboratories (Lytech laboratory and UNN) are summarized in Supplementary Table S1.

Quality control procedures

Both UNN, Department of Laboratory Medicine, and Lytech have internal and external quality control procedures that assure the reliability of the measurements of common clinical analytes. The external quality control procedures involve analysis of standard serum distributed in the country's network of laboratories participating in the program. Inter-assay coefficient of variation was calculated based on analyses of commercial control samples. UNN Department of Laboratory Medicine is reference laboratory for Northern Norway, accredited according to ISO 15189.

Data Analysis

Recalibration

Initially, we compared the biomarker measures from UNN and Lytech graphically by examining scatter plots and Bland–Altman plots (differential plots). Before further data analysis, outliers were excluded: observations >3 SDs from the mean difference were defined as outliers and removed (Supplementary Table S3). After exclusion of outliers, Pearson's correlation coefficient was computed and a Cusum test (Passing-Bablock) was performed to assess the linearity of relationship between UNN and Lytech values [4] . The Cusum test indicated a non-linear relationship between the two sets of biomarker measures for hsCRP and HbA1c. For those two analytes calibration equations were fitted separately in different ranges, with the break points determined using iterative procedure [5].

The calibration function for the relationship between split-sample measurements conducted in University Hospital of Northern Norway (UNN) Department of Laboratory Medicine and Lytech laboratory (Moscow) was determined using Deming regression, which accounts for errors in both the dependent and independent variables [6]. The regression equation UNN = Intercept+Slope*Lytech represents the regression relationship between paired values was assumed to be of the form UNN = Intercept+Slope*Lytech. Unweighted or weighted Deming regression methods were used in this calibration study.[9] The choice between the unweighted and weighted methods was made based on the distribution of the data points on the differential plot [6]. Weighted Deming regression was used if the coefficient of variation (CV) was constant while standard deviation changes proportionally to the concentration [6]. Statistical calculations were performed in R using the packages "mcr" (1.2.1), "VDSPCalibration" (1.0), and "segmented" (0.5-4.0).

Use of calibration study results

The resulting regression coefficients (intercept and slope) were used to recalibrate Know Your Heart study values so that they are comparable with Tromsø 7 study measurements. There is uncertainty in the estimation of the regression coefficients in the calibration models, which should be carried through to the subsequent analyses in which the recalibrated values are used in regression analyses. To account for this we used a "double-bootstrap" approach. This allows estimation of the confidence intervals for the regression coefficients in the main regression analyses (representing adjusted mean difference between recalibrated biomarker levels between the two studies), taking into account the uncertainty at both stages of the analysis by using bootstrapping for the calibration study sample and for the main study sample. The double bootstrap approach is described in more detail in Supplementary Methods M2 and we conducted a simulation study to demonstrate the validity of this approach for these purposes (Supplementary Methods M2).

Results:

Development and application of calibration equations

In general, the calibration study showed very good correlation between UNN Department of Laboratory Medicine values and Lytech values for most analytes (Supplementary Table S3). The exception was hs-cTnT, which showed Pearson's correlation of 0.883.

Also, the relationship between UNN Department of Laboratory Medicine values and Lytech values was linear for many analytes. The differential (Bland-Altman) plots and scatter plots are shown in Supplementary Figure 1. The regression equation coefficients are summarized in Supplementary Table S3. Departure from linearity was found for hsCRP and HbA1c. Therefore, different calibration equations were developed separately for each segment. The estimated break points for hsCRP are 1.45 mg/L and 5.57 mg/L, and for HbA1c - 7.48 %.

Because hs-cTnT test has high CV at low values [7], and its limit of quantification is at 13 ng/L it is not feasible to reliably calibrate this test as quantitative measure of Troponin T concentration. Therefore, values were compared above and below a threshold of top quintile (11 ng/L). Using the binary threshold, Lytech laboratory misclassified about 4 % of values relative to UNN Department of Laboratory Medicine.

Supplementary Table S3. The relationship between UNN Department of Laboratory Medicine values and Lytech values described via Pearson's R^2 and Deming regression.

Analyte	No of excluded outliers	R^2	Intercept, 95% CI	Slope, 95% CI
Total cholesterol, mmol/L	0	0.997	0.00 (-0.08, 0.08)	0.97 (0.96, 0.99)
HDL cholesterol, mmol/L	2	0.982	-0.20 (-0.26,-0.14)	1.17 (1.13, 1.22)
LDL cholesterol, mmol/L	2	0.986	-0.66 (-0.82,-0.50)	1.11 (1.07, 1.15)
Triglycerides, mmol/L	2	0.999	0.05 (0.03, 0.06)	0.99 (0.99, 1.00)
High sensitivity CRP, mg/L	1	0.996		
hsCRP, < 1.45 mg/L*			0.07 (0.05, 0.09)	0.70 (0.67, 0.73)
hsCRP, 1.445 - 5.57 mg/L*			0.35 (-0.40, -0.29)	0.96 (0.93, 0.98)
hsCRP, > 5.57 mg/L*			1.13 (0.74, 1.51)	0.68 (0.64, 0.73)
HbA1c, % (Glycated haemoglobin)	2	0.997		
HbA1c <7.48 %*			-0.99 (-1.37,-0.62)	1.22 (1.16, 1.30)
HbA1c >7.48 %*			0.63 (0.04, 1.222)	1.01 (0.95, 1.08)
Hs-cTnT, ng/L	0	0.883	-	-
Nt-proBNP, pg/mL	0	0.998	6.41 (4.69-8.13)	0.62 (0.59-0.64)

*Weighted Deming regression was used to develop calibration equations

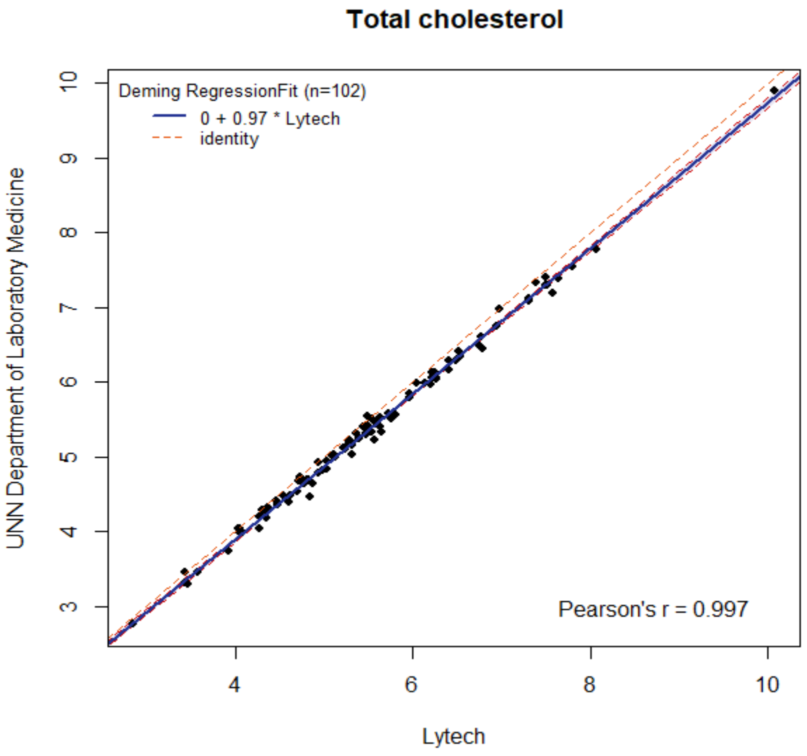

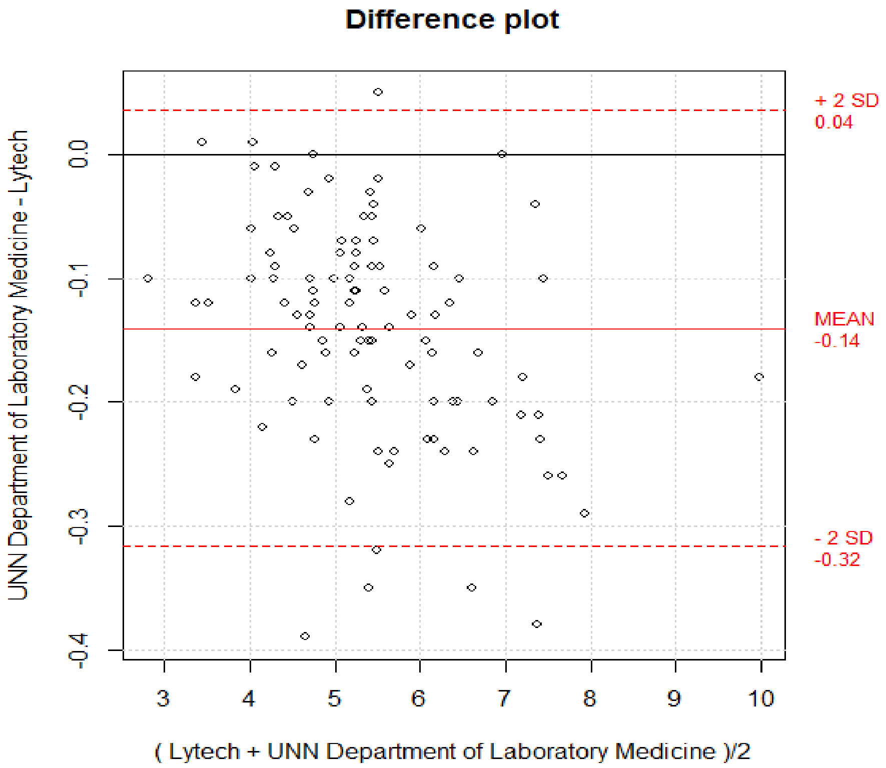

Supplementary Figure S1. Scatter and differential (Bland-Altman) plots of Lytech versus UNN assayed total cholesterol in the KYH recalibration subsample. Yellow dotted line on the scatter plot represents the identity line for measurements, while two red dotted lines represent the 95% Confidence Intervals for the biomarker values in the calibration sample.

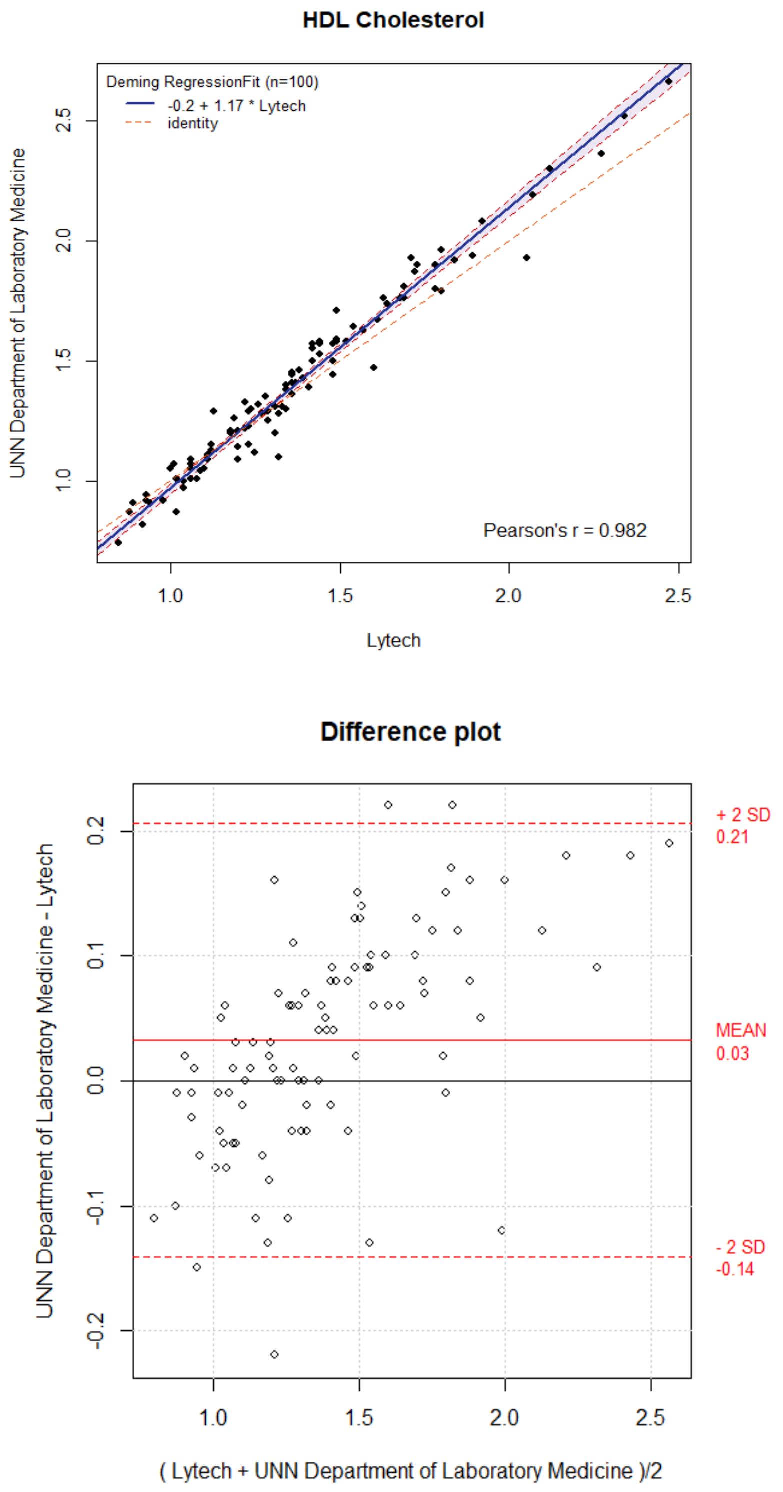

Supplementary Figure S2. Scatter and differential (Bland-Altman) plots of Lytech versus UNN assayed HDL-cholesterol in the KYH recalibration subsample

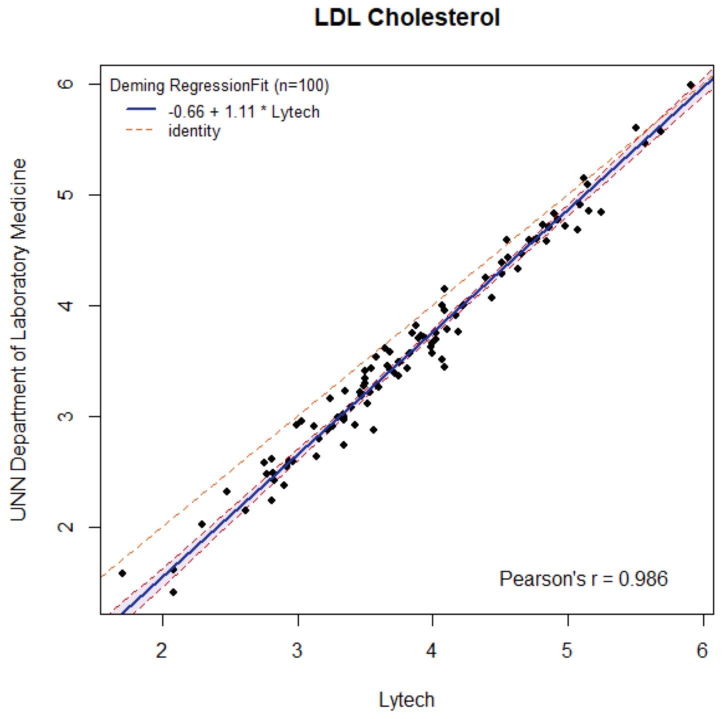

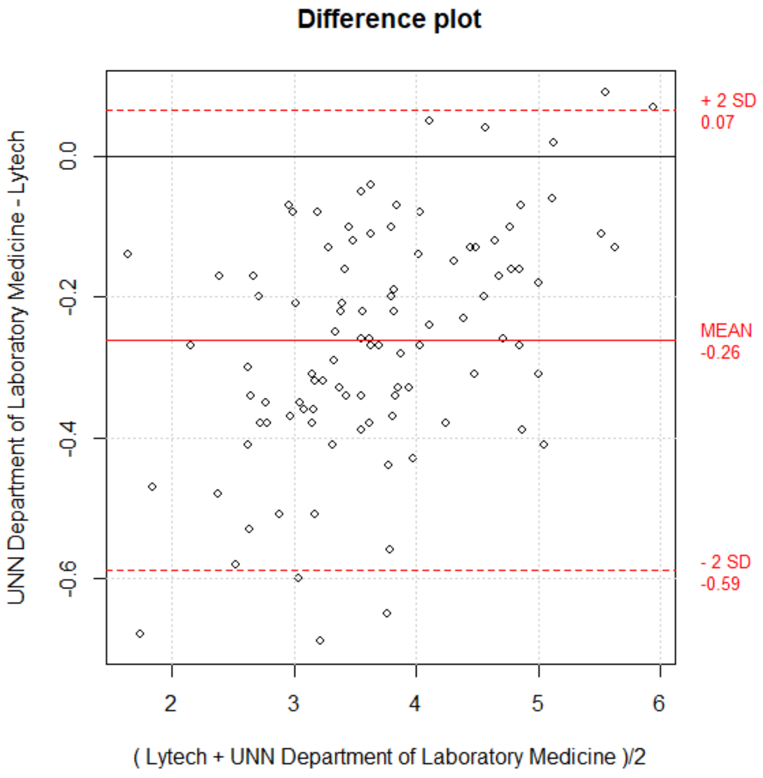

Supplementary Figure S3. Scatter and differential (Bland-Altman) plots of Lytech versus UNN assayed LDL-cholesterol in the KYH recalibration subsample

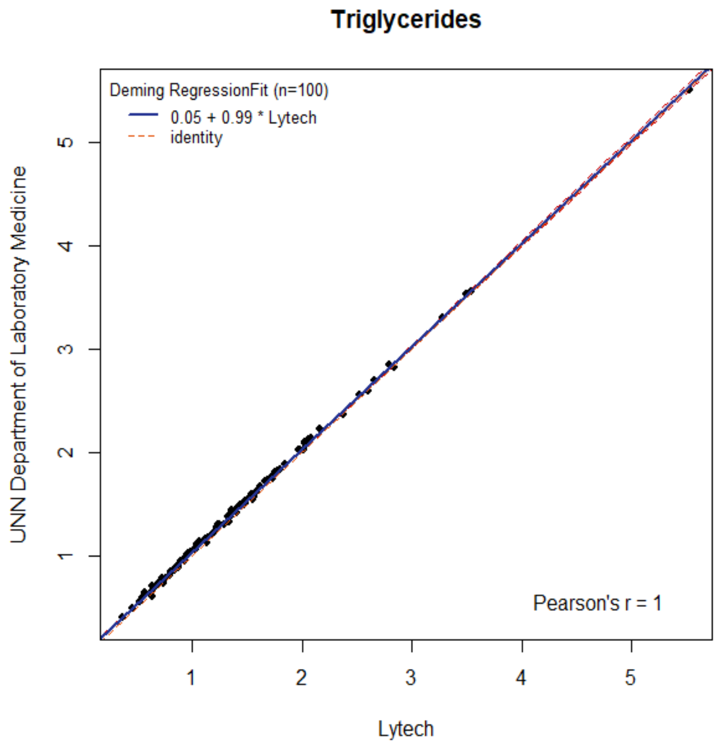

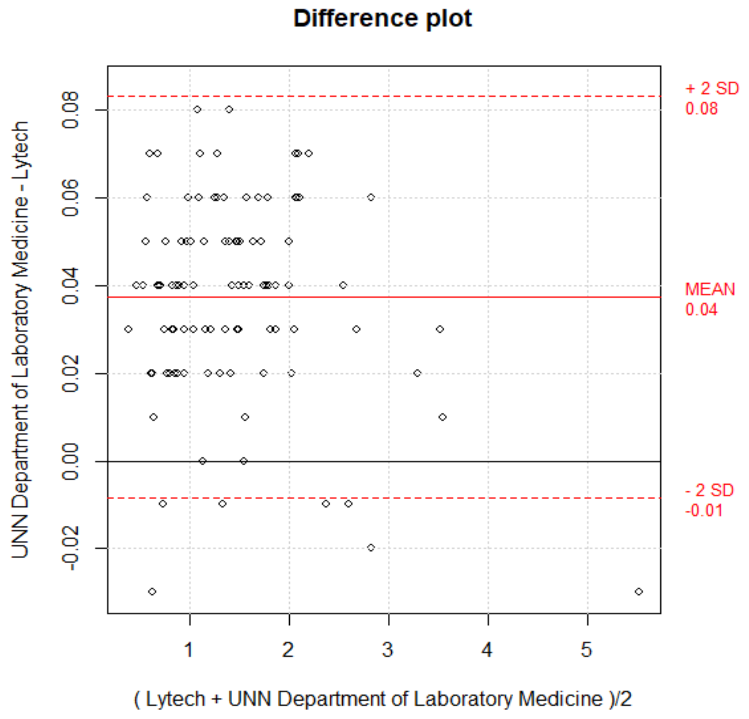

Supplementary Figure S4. Scatter and differential (Bland-Altman) plots of Lytech versus UNN assayed tryglycerides in the KYH recalibration subsample

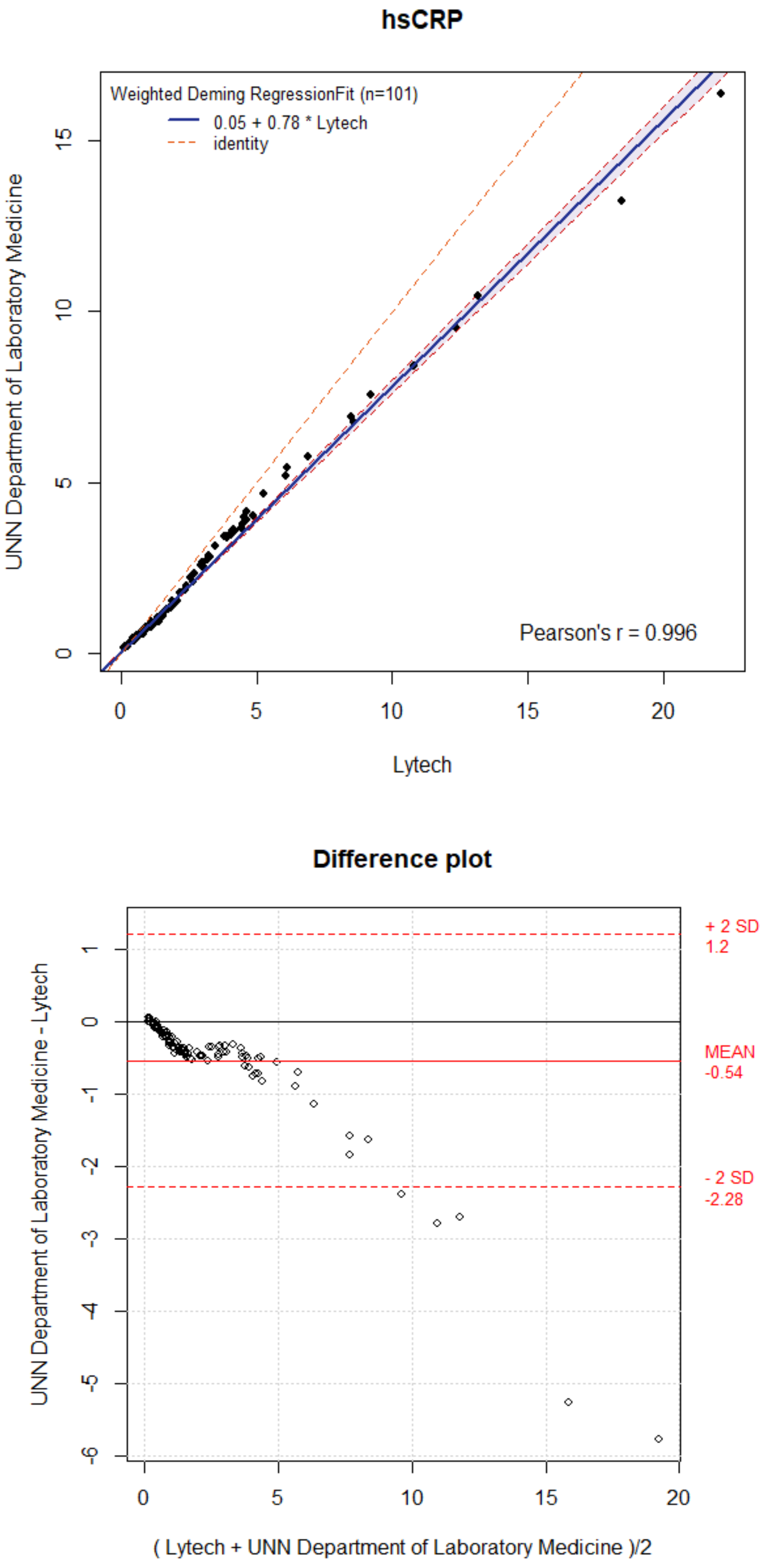

Supplementary Figure S5. Scatter and differential (Bland-Altman) plots of Lytech versus UNN assayed hsCRP in the KYH recalibration subsample.

A) hsCRP < **1.45** mg/L

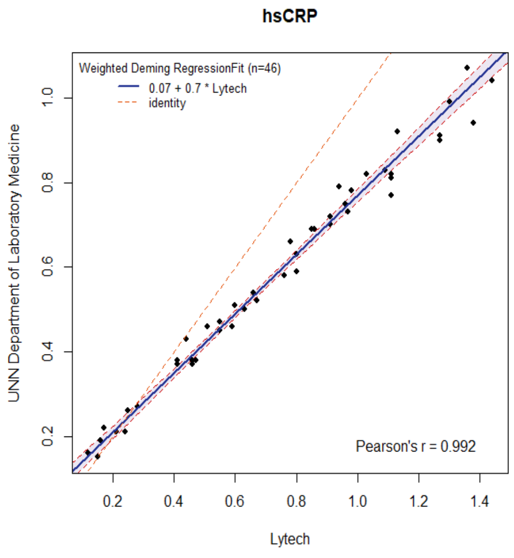

B) hsCRP 1.45 - 5.57 mg/L

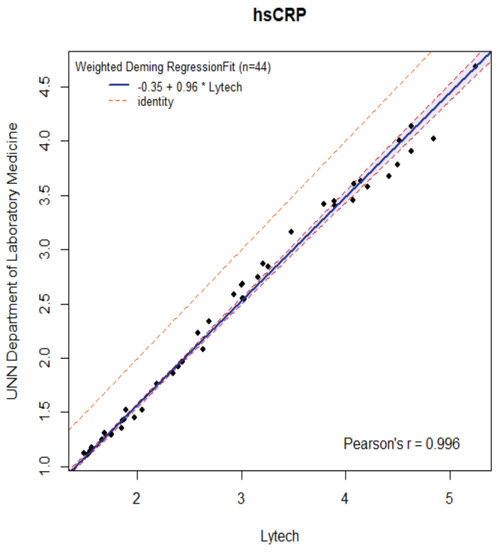

C) hsCRP > **5.57** mg/L

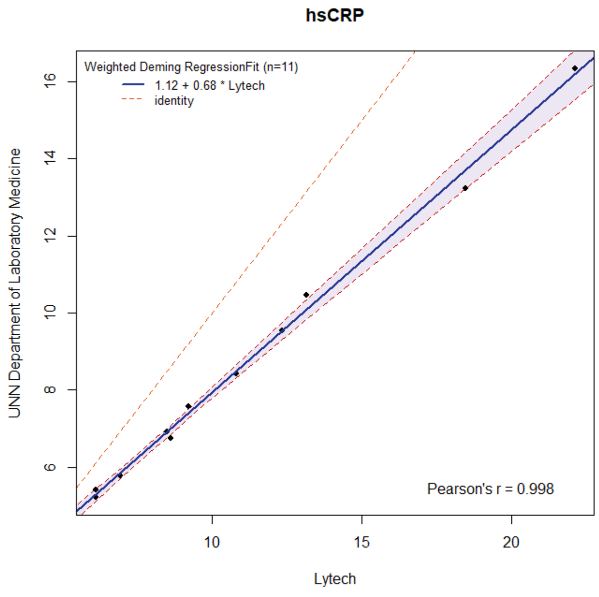

Supplementary Figure S6. Scatter plots of Lytech versus UNN assayed hsCRP in the KYH recalibration subsample with regression line split into three segments. A) hsCRP < 1.45 mg/L; B) hsCRP 1.45 - 5.57 mg/L; C) hsCRP > 5.57 mg/L

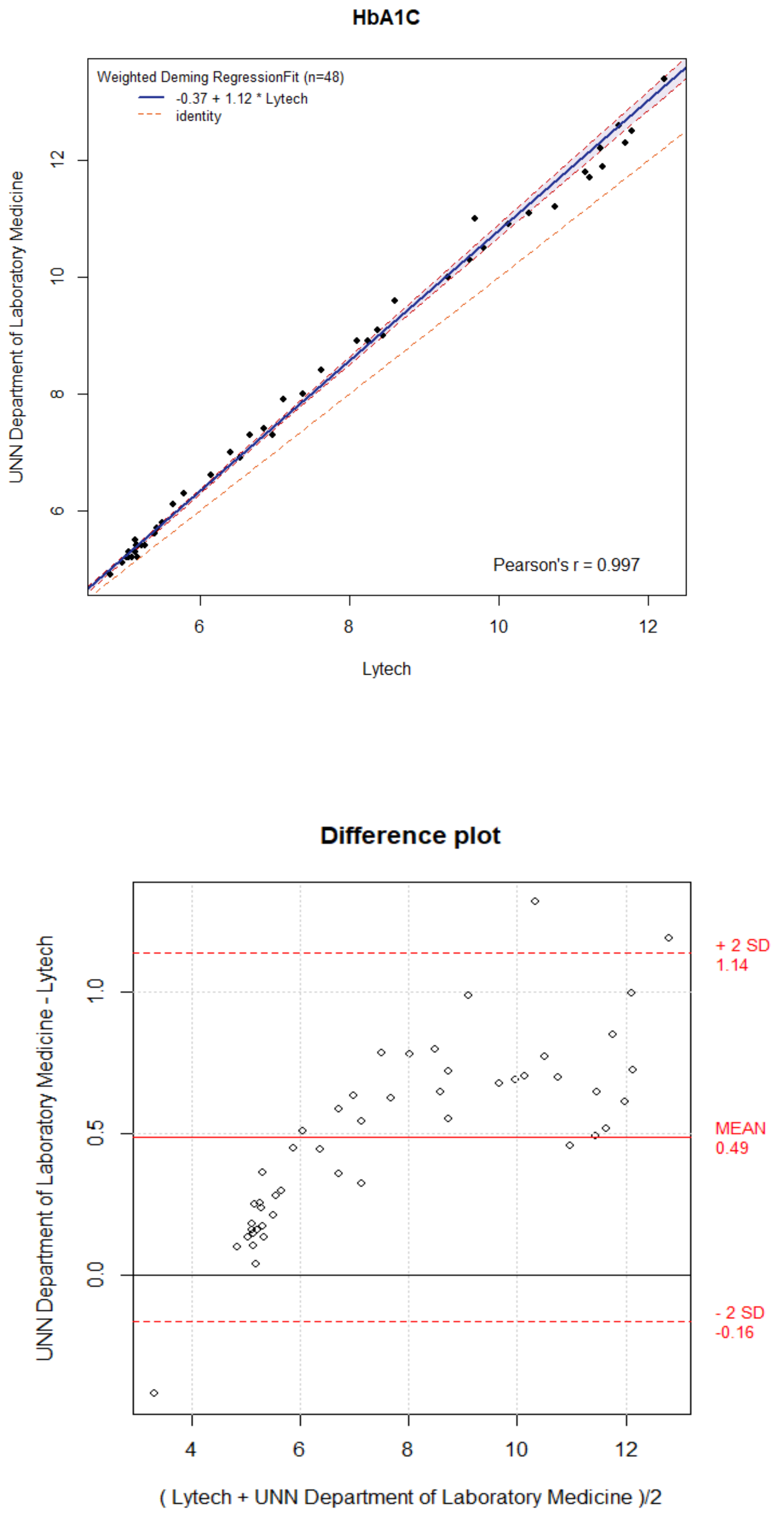

Supplementary Figure S7. Scatter and differential (Bland-Altman) plots of Lytech versus UNN assayed HbA1c in the KYH recalibration subsample

A) HbA1C < 7.48 %

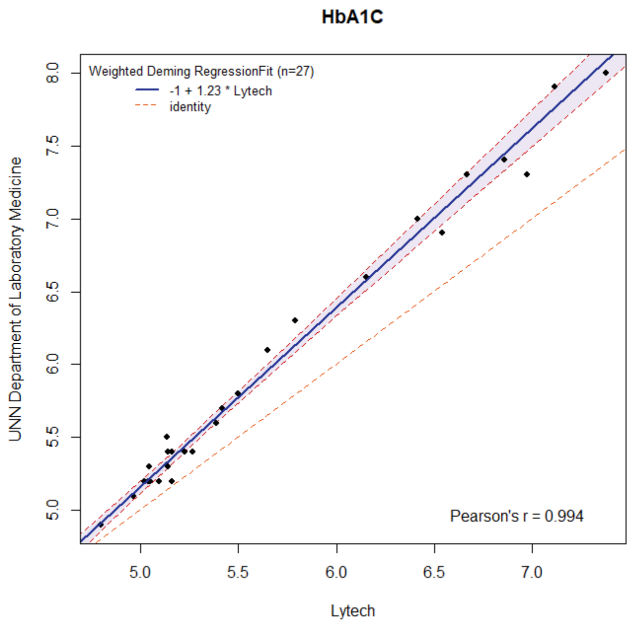

B) HbA1C > 7.48 %

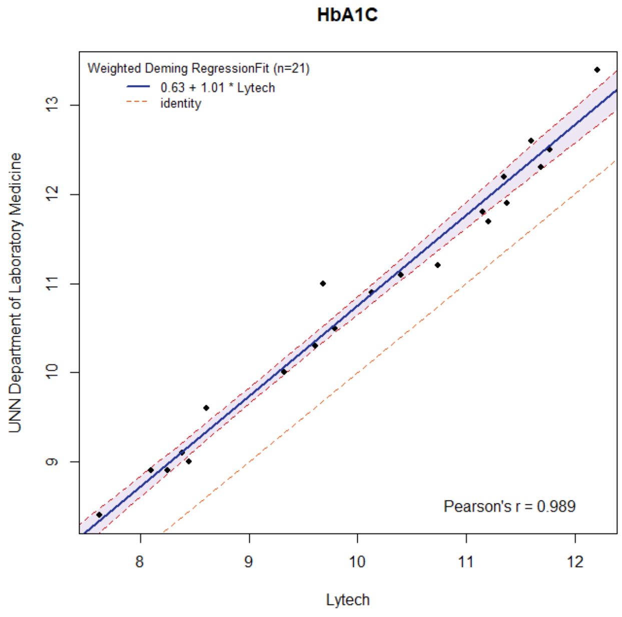

Supplementary Figure S8. Scatter and differential (Bland-Altman) plots of Lytech versus UNN assayed HBA1c in the KYH recalibration subsample with regression line split into two segments: A) HbA1c < 7.48 %; B) HbA1c > 7.48 %

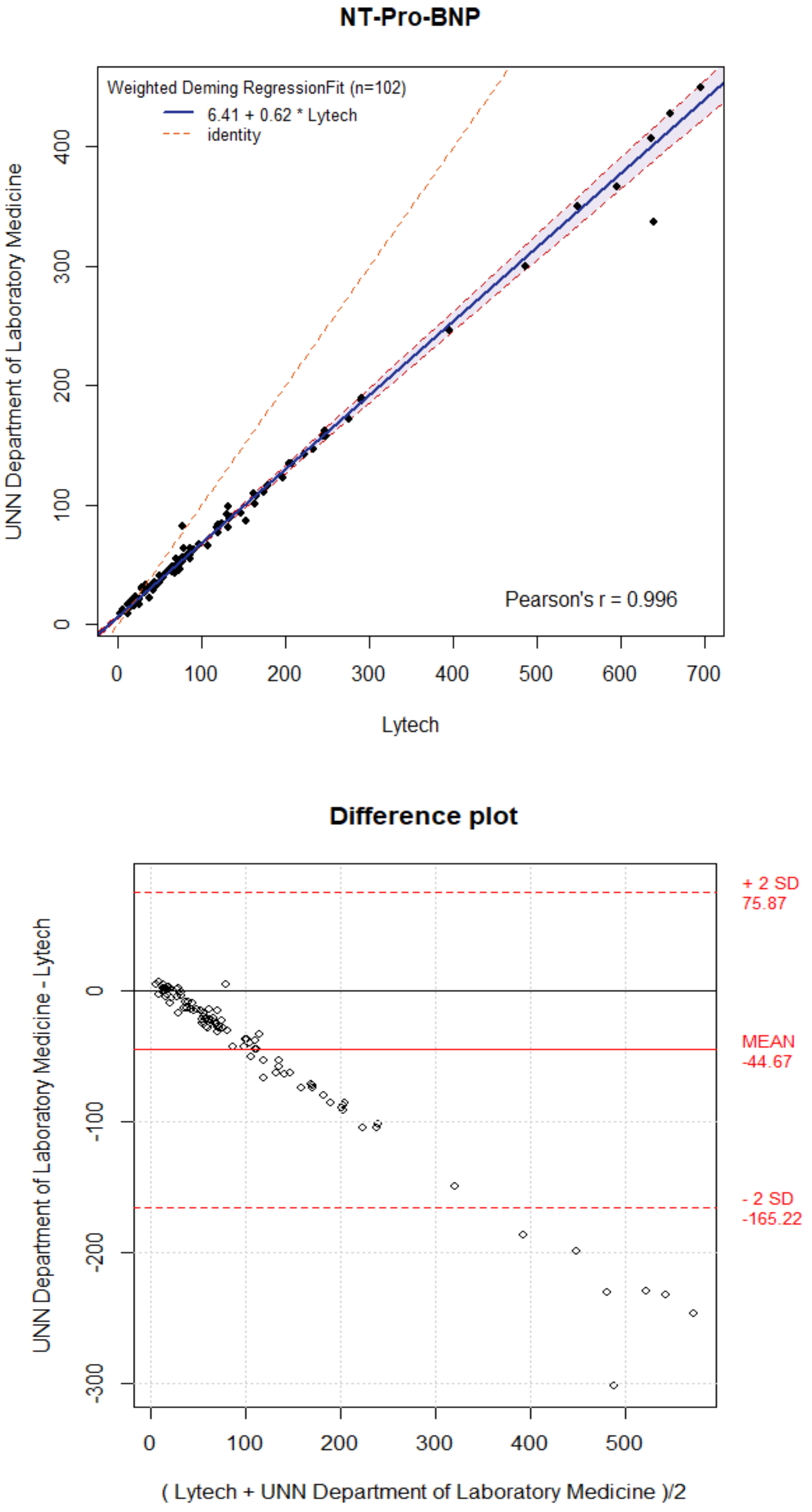

Supplementary Figure S9. Scatter and differential (Bland-Altman) plots of Lytech versus UNN assayed NT-proBNP in the KYH recalibration subsample

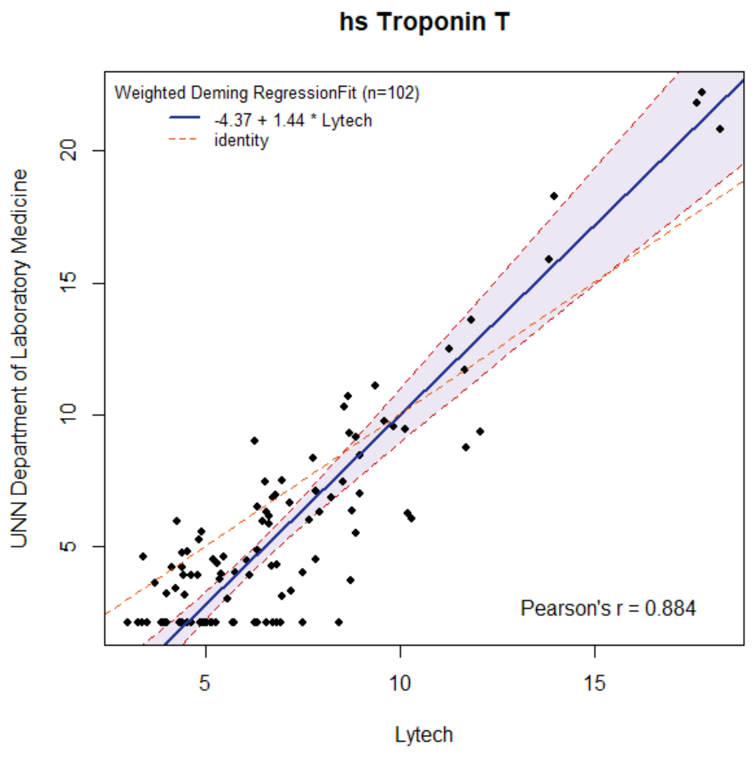

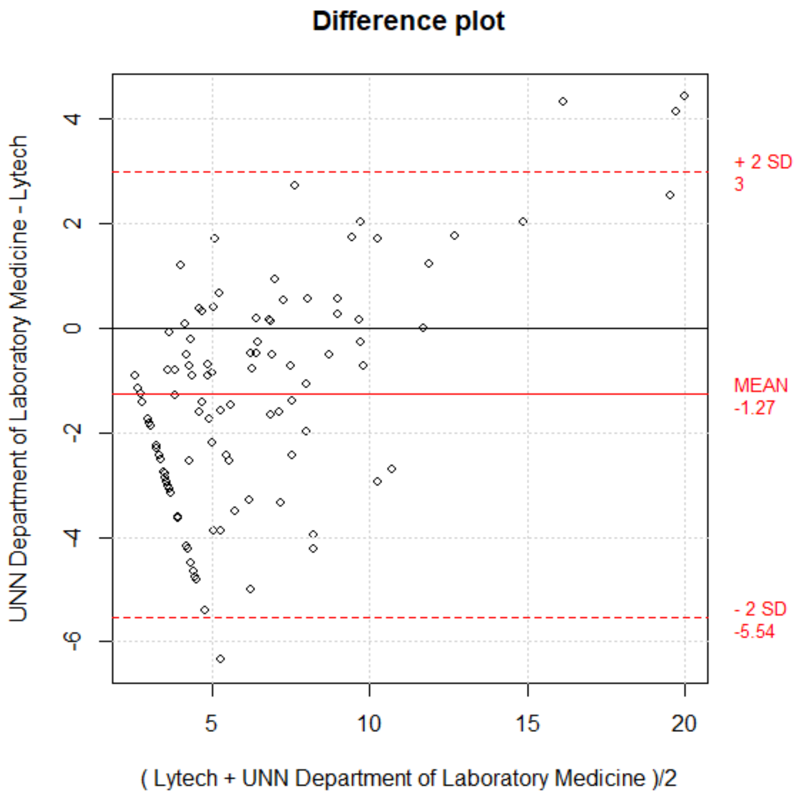

Supplementary Figure S10. Scatter and differential (Bland-Altman) plots of Lytech versus UNN assayed hs-cTnT in the KYH recalibration subsample

Supplementary Table S4. Concordance of hs-cTnT measurements in UNN Department of Laboratory Medicine and Lytech laboratory by threshold of 11 ng/L and 8.07 ng7L

hs-cTnT	UNN Department of Laboratory Medicine	
Lytech laboratory		
	< 11 ng/L	> 11 ng/L
< 11 ng/L, N (%)	93 (95.9%)	4 (4.1%)
> 11 ng/L, N (%)	0 (0%)	5 (100%)
	< 8.07 ng/L	> 8.07 ng/L
< 8.07 ng/L, N (%)	81 (92.1)	7 (7.9)
> 8.07 ng/L, N (%)	0	14 (100)

Use of calibration study results and limitations

The calibration function/equation represents the systematic bias in the KYH measurements relative to the Tromsø 7 measurements, due to the measurements being made in two different laboratories (Supplementary Table S5). The slope in the calibration equation represents the proportional error, and the intercept represents the constant error. Further statistical analysis involving comparisons of two populations using KYH and Tromsø 7 data should use the calibrated values. For example, when one is interested in comparing total cholesterol levels in Know Your Heart and Tromsø 7, it is important to remove the difference due to differences in analytic procedures in two laboratories.

However, although the appropriate calibration can account for the systematic bias, the recalibrated values have some uncertainty because the regression coefficients in the calibration equation are estimated (using the calibration study data) rather than being known exactly. This uncertainty should be accounted for in subsequent analyses using the recalibrated values for valid statistical inference. It is possible to do that by calculating confidence intervals of the estimates using a double-bootstrap procedure (Supplementary Material S2).

Due to appreciable imprecision of hs-cTnT assays at the low values seen in the general population, the development of calibration equation for this biomarker was not possible. Therefore, we compared hs-cTnT values between UNN Department of Laboratory Medicine and Lytech laboratory by binary threshold. The high values of hs-cTnT in Lytech are also classified as high in UNN, and low values are mostly (95.9%) also classified as low in UNN (Supplementary Table S4). Therefore, if further analysis of hs-cTnT is planned which involves both Tromsø 7 and Know Your Heart data, it should include the sensitivity analysis using with hs-cTnT as a binary variable.

Supplementary Table S5. Recalibration recommendations to maximize comparability of laboratory assays in Know Your Heart and Tromsø 7 study.

	Mean*			
	Lytech	UNN	Recommended calibration equation**	Comment
Total cholesterol, mmol/L	5.50	5.36	0 + 0.97*KYH	Small proportional bias, UNN measures lower
HDL cholestrol, mmol/L	1.38	1.41	-0.2 + 1.17*KYH	
LDL cholesterol, mmol/L	3.82	3.56	-0.66 + 1.11*KYH	UNN values are lower
Triglycerides, mmol/L	1.41	1.45	0.05 + 0.99*KYH	
High sensitivity CRP, mg/L	2.84	2.30	***<1.45 mg/L:***<br>0.07+ 0.7*KYH<br><br>***1.45 - 5.569 mg/L:***<br>-0.35 + 0.96*KYH<br><br>***>5.57 mg/L:***<br>1.12 + 0.68*KYH	The recalibration of KYH values should be done using 3 equations for 3 segments: <1.445 mg/L, 1.445 - 5.569 mg/L, >5.569 mg/L.
HBA1c, % (Glycated haemoglobin)	7.5	8.00	***<7.48 %***<br>-0.99 + 1.22*KYH<br><br>***>7.48 %***<br>0.63+1.01*KYH	The recalibration of KYH values should be done using 2 equations for 2 segments: <7.48 %, >7.48 %
hs-cTnT, ng/L	7.00		Not applicable	The analysis of hs-cTnT data should be performed using threshold of top quantile of biomarker distribution, recalibration as a quantitative variable is not possible.
NT-proBNP, pg/mL	132.2	87.6	6.41 + 0.62*KYH	UNN measures lower

*Means are after exclusion of outliers, UNN Department of Laboratory Medicine

**Recommendation should be applied to Know Your Heart sample if the comparisons with Tromsø 7 study are planned

Supplementary Methods 2

Simulation Study

Aims:

1) to assess whether the "double-bootstrap" method for obtaining standard errors and confidence intervals for the mean differences based on recalibrated biomarker measurements is correct;
2) to compare the results obtained with "double-bootstrap" method with standard method which ignores uncertainty in the estimation of the regression coefficients in the calibration model;

Data-generating mechanisms:

Three samples will be simulated:

(1) "Study A" – a sample of size N_A. Biomarker values are simulated from a Normal distribution with mean E(A) and standard deviation SD(A);

(2) "Study B" – a sample of size N_B. Biomarker values are simulated from a Normal distribution with mean E(B) and standard deviation SD(B).

(3) External calibration sample – a sample size N_c with paired data (x, y); where x is corresponds to measurements by the instrument used in the Study A and y corresponds to measurements by the instrument used in the Study B. Paired biomarker values (x, y) are generated, with x generated from a normal distribution with mean E(x) and standard deviation SD (x), and y generated from a conditional normal distribution with mean b0 +b1*x and standard deviation s, i.e. y= b0 +b1*x + e, where e is normally distributed with mean 0 and standard deviation s. Both values in the validation sample are assumed to be error-prone

measures of an underlying true value: variable x has measurement error CVx and variable y has measurement error CVy (error ratio = CV_1^2/CV_2^2).

The main analysis uses data from studies A and B. Study B is considered as the reference study for the purposes of calibration. The real mean difference in biomarkers level between Study A and Study B is E(Ac)- E(B), where Ac denotes the recalibrated biomarker values obtained using the calibration coefficients obtained in the validation sample: b0 and b1.

Several scenarios were assessed in this simulation study, those are selected according to the characteristics of the real data that are available to researcher and need to be analysed (Supplementary Table S6).

Supplementary Table S6. Scenarios for the simulation study based on eight biomarkers of interest.

	Scenario							
Parameter	1	2	3	4	5	6	7	8
N_A	1700	1700	1700	1700	1700	1700	1700	1700
N_B	8302	8302	8302	8302	8302	2712	8302	8302
E (A)	5.42	1.31	3.69	1.70	94.59	0.97	5.42	5.48
E (B)	5.46	1.37	3.69	1.78	81.76	1.04	5.46	5.60
SD (A)	1.14	0.33	0.91	1.38	28.10	0.37	1.14	0.48
SD (B)	1.04	0.39	0.97	1.12	14.37	0.15	1.04	0.40
E(Ac)-E(B)	-0.20	-0.04	-0.25	-0.04	3.16	-0.01	-0.20	0.095
N_c	100	100	100	100	100	100	500	25
E(x)	5.36	1.41	3.55	1.45	76.03	0.91	5.36	5.99
SD(x)	1.14	0.39	0.92	0.79	14.27	0.16	1.14	0.94
b0	0	-0.2	-0.66	0.05	-29.42	0.06	0	-0.99
b1	0.97	1.17	1.11	0.99	1.21	1	0.97	1.22
s	0.0817,	0.0736,	0.1516,	0.0224,	2.695,	0.0194	0.0817	0.1069
CV_1^2/CV_2^2	2.1	2.0	30.6	16.7	13.8	2.7	2.1	1.7

Methods:

I. Standard

The calibration coefficients are estimated based on simulated validation study using Deming regression. The Study A simulated values are multiplied by the calibration coefficients (b0 and b1). The calibrated values in simulated dataset are regressed on the variable "Study" to estimate the mean difference, standard error of the difference (SE), and 95% confidence interval (95% CI).

II. "Double bootstrap"

The steps are as follows:

(i) Take M random samples with replacement from the calibration study data.

(ii) Perform the Deming regression analysis for each of the M calibration study samples, to obtain M sets of estimates of the calibration model parameters (intercept and slope).

(iii) Take M random samples with replacement within the main study data, with the sampling being stratified by study (A and B).

(iv) In bootstrap sample m ($m = 1, \ldots, M$) from the main study, use the calibration model parameters from bootstrap sample m of the calibration study data to obtain recalibrated biomarker measures in study A.

(v) In sample m ($m = 1, \ldots, M$) of the main study, the calibrated biomarker values regress on the variable "Study" to obtain the mean difference. The standard deviation of the B estimates provides an estimate of the standard error for the mean difference, and the 2.5% and 97.5% percentiles of B estimates gives the percentile-based bootstrap 95% confidence intervals.

We used B=500 and the simulation was repeated 1000 times.

Estimands:

We focus on the standard error for the mean difference and coverage of the confidence intervals of the mean difference between Study A and Study B obtained using the standard approach and the proposed double-bootstrap method.

Performance measures:

The "true" standard error (empirical standard error, EmpSE) is estimated by the standard deviation of the 1000 estimates of the mean difference. The means of the standard errors (ModSE) obtained with 1000 repetitions of standard (ModSE) and double-bootstrap methods (BootSE) are compared with the true standard error. Coverage of the confidence intervals obtained with the bootstrapping approach and the standard approach is given by the percentage of 95% confidence intervals for the mean difference (over the 100 simulations) that contain the true mean difference. Results are shown in Supplementary Table S7.

Supplementary Table S7. Results from simulation study: Estimates of the performance measures of interest for the range of scenarios.

Scenarios	EmpSE*	ModSE* (standard)	BootSE* (bootstrap)	Coverage (%) (standard)	Coverage (%)(bootstrap)
1	0.032	0.028	0.030	93	95
2	0.013	0.010	0.013	83	90
3	0.032	0.026	0.031	89	94
4	0.035	0.031	0.035	92	95
5	0.983	0.517	0.972	66	92
6	0.012	0.009	0.012	87	94
7	0.028	0.028	0.029	94	95
8	0.029	0.012	0.029	56	94

*EmpSE – Empirical standard error; ModSE – Model standard error; BootSE – bootstrap standard error

In all explored scenarios standard error obtained using double-bootstrap procedure is close to the empirical standard error. However, the standard error obtained using standard procedure is smaller than the empirical standard error, indicating that the standard approach underestimates the uncertainty in the estimated mean difference. The underestimation is severe in some scenarios, for example when the validation sample is small. Similarly, the coverage of

percentile-based bootstrap confidence intervals is close to the nominal level of 95% while under-coverage is observed when confidence intervals are obtained using standard method, with the under-coverage again being severe in some scenarios. The standard approach would be expected to improve as the size of the calibration study increases. However, in a situation of calibration samples of realistic (i.e. relatively small) sample size it is appropriate to estimate standard errors and confidence intervals using the proposed “double bootstrap” approach.

Supplementary Table S8. Age-standardized proportion of participants with hs-cTnT value above top quantile of distribution in KYH and Tromsø 7.

	KYH	Tromsø 7	p-value
	Men		
Hs-cTnT > 11.0 ng/L	0.19 (0.17, 0.21)	0.12 (0.09, 0.15)	<0.001
	Women		
Hs-cTnT > 8.07 ng/L	0.22 (0.20, 0.23)	0.06 (0.03, 0.08)	<0.001

Supplementary Table S9. The odds of hs-cTnT being in the top quantile of distribution in KYH study compared to Tromsø 7, explained by adjustment for classical CVD risk factors: smoking, BMI, WHR, SBP and DBP, diabetes, education.

	N	Model 1 (adjusted for age) OR (95% CI)	Model 2 (adjusted for age, smoking, BMI, WHR, SBP, DBP, diabetes, education) OR (95% CI)
Men			
Hs-cTnT > 11 ng/L	2196	1.95 (1.5, 2.52)	1.88 (1.41, 2.52)
Women			
Hs-cTnT > 8.07 ng/L	2881	5.93 (4.34, 8.1)	4.85 (3.4, 6.91)

References

1 Parrinello CM, Grams ME, Couper D, *et al.* Recalibration of blood analytes over 25 years in the atherosclerosis risk in communities study: impact of recalibration on chronic kidney disease prevalence and incidence. *Clin Chem* 2015;**61**:938-47.

2 Stepman HC, Tiikkainen U, Stockl D, *et al.* Measurements for 8 common analytes in native sera identify inadequate standardization among 6 routine laboratory assays. *Clin Chem* 2014;**60**:855-63.

3 Selvin E, Coresh J, Zhu H, *et al.* Measurement of HbA1c from stored whole blood samples in the Atherosclerosis Risk in Communities study. *Journal of diabetes* 2010;**2**:118-24.

4 Passing H, Bablok W. A new biometrical procedure for testing the equality of measurements from two different analytical methods. Application of linear regression procedures for method comparison studies in clinical chemistry, Part I. *Clinical chemistry and laboratory medicine* 1983;**21**:709-20.

5 Muggeo VM. Estimating regression models with unknown break-points. *Statistics in medicine* 2003;**22**:3055-71.

6 Linnet K. Estimation of the linear relationship between the measurements of two methods with proportional errors. *Statistics in medicine* 1990;**9**:1463-73.

7 Egger M, Dieplinger B, Mueller T. One-year in vitro stability of cardiac troponins and galectin-3 in different sample types. *Clinica chimica acta; international journal of clinical chemistry* 2018;**476**:117-22.
